# Identification of Candidate Gene Controlling Soluble Sugar Degradation During Postharvest Storage of Sweet Corn Based on BSA-Seq

**DOI:** 10.3390/genes17030291

**Published:** 2026-02-27

**Authors:** Mengyun Ren, Meixing Wang, Dong Wang, Yifeng Huang, Longgang Du

**Affiliations:** Institute of Crop and Nuclear Technology Utilization, Zhejiang Academy of Agricultural Sciences, Hangzhou 310021, China; renmengyun@zaas.ac.cn (M.R.); wangmx@zaas.ac.cn (M.W.); wangdong@zaas.ac.cn (D.W.); hyfeng302@163.com (Y.H.)

**Keywords:** sweet corn, postharvest shelf life, soluble sugar degradation, bulked segregant analysis sequencing, candidate gene

## Abstract

**Background/Objectives**: Sweetness is a key determinant of the eating quality of sweet corn, primarily governed by the soluble sugar content in kernels. The soluble sugar content decreases rapidly during the postharvest shelf life, which directly affects the flavor and quality. Relatively few studies have been conducted on the shelf life of sweet corn. **Methods**: An F_6_ recombinant inbred line (RIL) population was constructed from two super sweet inbred lines with contrasting soluble sugar degradation rates: D174 (low degradation rate) and D179 (high degradation rate). Extreme phenotype pools were established using soluble sugar content as the target trait. Based on bulked segregant analysis sequencing, we identified chromosomal segments associated with postharvest soluble sugar reduction in sweet corn, annotated the gene information within these segments, and analyzed the functions of the annotated genes using the Gene Ontology and Genomes databases. **Results**: Results revealed three associated regions located at 44,205,775–45,290,843 bp on chromosome 4, 6,250,656–6,744,665 bp on chromosome 2, and 135,428,709–136,732,132 bp on chromosome 10. This interval contained 195 genes. Integrated analysis of gene expression, gene annotations, and quantitative real-time PCR indicated that *Zm00001eb069070*, which is highly expressed in kernels with a prolonged shelf life, might be a key candidate gene regulating soluble sugar degradation in sweet corn. **Conclusions**: This study provides valuable genetic resources for the improvement of favorable agronomic traits and the advancement of molecular breeding strategies for sweet corn.

## 1. Introduction

Sweet corn is a maize variant derived from mutations in genes involved in sugar conversion within the endosperm, such as *su1*, *sh2*, *bt2*, and *se* [1,2]. This impairs starch synthesis and leads to a substantial accumulation of soluble sugars [1]. Depending on the variations in these causal genes, sweet corn can be categorized into different genetic types (e.g., normal sweet corn, enhanced sweet corn, and super sweet corn), with distinct quality traits among these types [3]. China has the world’s largest sweet corn cultivation area, covering approximately 5.3 million hectares [4]. Favored by consumers for its high sugar content, abundant antioxidants [5,6], vitamins [7,8], and mineral elements [9], sweet corn has become a globally important horticultural crop for fresh onsumption and processing [10], with an annual farm-gate transaction value of $1.4 billion (74% fresh market, 26% processed market including canned and frozen products).

Eating quality is a critical criterion for evaluating sweet corn varieties. Sweetness is a key index of the eating quality of sweet corn and is directly determined by the compositions and contents of soluble sugars, compared to insoluble sugars such as starch [11]. Soluble sugars primarily comprise fructose, sucrose, and glucose. Fructose possesses the greatest perceived sweetness, being about 1.73 times sweeter than sucrose and 2.34 times sweeter than glucose [12]. Sucrose, a disaccharide composed of glucose and fructose, offers a well-rounded and stable sweetness profile and is widely used in cooking and baking [13]. Plants have evolved a cyclic metabolic system centered around sucrose metabolism to regulate sugar homeostasis, involving three key sugar metabolic enzymes: invertase (Inv), sucrose phosphate synthase (SPS), and sucrose synthase (SUS) [14,15]. Specifically, sucrose is hydrolyzed by Inv into glucose and fructose, which are phosphorylated by fructokinase (FRK) and hexokinase (HXK), respectively, to form fructose-6-phosphate (F6P) and glucose-6-phosphate (G6P); SUS reversibly converts sucrose into UDP-glucose and fructose [16,17]; UDP-glucose and F6P can be converted into sucrose by SPS and sucrose-phosphate phosphatase (SPP) [18]. Other sugars, such as starch or sorbitol, can also be associated with sucrose metabolism through enzyme catalysis, such as starch synthase, β-amylase, sorbitol dehydrogenase, and sorbitol oxidase. These sugar pathways also involve various transport enzymes and calcium-dependent protein kinases [19].

Previous studies revealed that sweet corn mainly had sucrose, fructose, and glucose, with sucrose (accounting for over 70% of total soluble sugars) as the predominant component. The contents of fructose and glucose are approximately 12% and 17%, respectively [20]. After harvesting at the milk stage, the balance of soluble sugar is disrupted, and fresh corn nutritional traits change dynamically [21,22]. The high-water content in sweet corn causes intense respiration and promotes the conversion of sucrose to starch, leading to a reduction in sweetness and eating quality [23,24,25]. The consumption of nutrients, water loss, and a rapid decline in the quality and flavor of ears during storage shorten the shelf life and reduce their commercial value [26,27]. Therefore, extending the effective storage life of sweet corn is crucial for enhancing its commercial value. Low-temperature storage is the most commonly used method for preserving sweet corn [28,29]. Nevertheless, low temperatures only slow the rate of sucrose degradation while increasing refrigeration costs and reducing economic benefits, thus failing to solve the problem fundamentally. Current understanding of the mechanisms underlying postharvest eating quality decline in sweet corn is primarily based on physiological data [30], with limited molecular evidence derived mainly from integrated omics studies, such as transcriptomics and metabolomics [29,31]. In some sweet corn lines, total sugar content may increase during storage due to the conversion of starch to soluble sugars via amylase-mediated degradation [32]. This phenomenon has been linked to enhanced α-amylase activity in certain genotypes with low sugar degradation rates. Wang et al. [33] reported that a novel endosperm mutant, *bt1774*, restricted starch synthesis, reduced starch content, and significantly increased soluble sugar content. These findings suggest that genetic improvement strategies targeting starch-sugar metabolism can be exploited to enhance flavor and extend the shelf life of sweet corn [34]. Therefore, investigating the molecular mechanisms underlying the postharvest sweetness decline in sweet corn and addressing the problem of deteriorating eating quality are important for developing postharvest preservation technologies and extending the shelf life of sweet corn.

Bulked segregation analysis (BSA) is an effective approach for rapidly identifying molecular markers linked to target genes by constructing mixed pools of individuals with extreme phenotypes [35]. The integration of BSA with RNA-seq for trait mapping has emerged as a rapid, accurate, and efficient analytical strategy, and it has been widely applied in various crops such as rice [36], cauliflower [37], cucumber [38], tomato [39], peach [40], and maize [41]. However, there are relatively few studies on the application of BSA in maize sugar metabolism, and no relevant reports have been found regarding its use in dissecting the postharvest sugar metabolism of fresh corn. The postharvest sweetness decline in sweet corn is a complex trait influenced by kernel structure, environmental factors, and chemical components. Currently, the causes and mechanisms underlying the deterioration of food quality during storage are unclear. Most previous studies have focused on the detection of basic physicochemical properties, with few functional genes being reported. To further explore the genes related to sugar metabolism in sweet corn, we constructed an F_6_ population using the inbred line D179 (a super sweet material with a high-degradation rate) as the female parent and D174 (a super sweet material with a low-degradation rate) as the male parent. Extreme phenotype pools were established using soluble sugar content as the target trait. The objectives of this study were to (1) map candidate intervals controlling postharvest soluble sugar degradation in sweet corn via bulked segregation analysis sequencing (BSA-seq), (2) screen candidate genes associated with postharvest soluble sugar degradation, and (3) analyze the genetic basis of this trait and clarify the postharvest sugar metabolic pathway and its variations. This study provides a theoretical foundation for improving postharvest storage and processing technologies for sweet corn, as well as for facilitating the molecular-assisted breeding of new sweet corn varieties with prolonged shelf life.

## 2. Materials and Methods

### 2.1. Plant Materials

D179 is a super sweet inbred line with a high soluble sugar degradation rate, while D174 is a super sweet inbred line with a low degradation rate. These two inbred parental lines were hybridized with the combinations of D179 × D174, and an F_6_ recombinant inbred line (RIL) population was obtained, comprising 204 families by simultaneous self-breeding. Three lines in the F_6_ RIL population were selected for further validation, including R218 (low degradation), R311 (moderate degradation), and R254 (high degradation). All the parental lines and population were independently developed and selected by Zhejiang Academy of Agricultural Sciences.

### 2.2. Growth Conditions and Experimental Design

The experiment was conducted at the experimental base of the Zhejiang Academy of Agricultural Sciences (Hangzhou, China). Each genotype was planted in two rows, with 15 plants per row, a row spacing of 60 cm, and a plant spacing of 30 cm. Cultivation and management practices were consistent with those of conventional cornfields. To prevent cross-pollination with foreign pollen, all plants were subjected to artificial bagging before pollination, and the ears used in the experiments were obtained exclusively from self-pollinated plants with bagging. All ears were harvested at 19 days after pollination (DAP) and subsequently stored at (25 ± 1) °C with approximately 50% ± 5% relative humidity for 8 and 72 h postharvest without any packaging or additional treatment. Samples collected at 0 h (immediately after collection) were directly frozen in liquid nitrogen. The middle 3–5 cm of kernels were collected from 15 uniform ears per line. For each storage duration (0, 8, and 72 h), kernels were sampled with three biological replicates per time point, and each replicate consisted of kernels from 3 to 5 ears. All samples were stored at −80 °C. All harvested ears were free of pest and disease damage.

### 2.3. Determination of Soluble Sugar Content

Fresh weight, water content, and soluble sugar content were determined for each population. Based on previous studies [20], samples were collected at three time points: 0, 8, and 72 h postharvest. Soluble sugar content was determined using a modified high-performance liquid chromatography–evaporative light scattering detection (HPLC-ELSD) method developed by our research group [42]. Thirty ears were randomly harvested from each material, with three replicates per sample.

### 2.4. Bulked Segregant Analysis Sequencing

#### 2.4.1. DNA Extraction, Library Construction, and Sequencing

For bulked segregation analysis, fresh kernels were collected and immediately frozen in liquid nitrogen from individuals consisting of two parents (D179 and D174). Based on soluble sugar degradation rate, 30 lines with stable low sugar degradation rates and 30 lines with stable high sugar degradation rates were selected to construct extreme phenotype pools (low- and high-degradation pools). Genomic DNA was extracted from each selected line and mixed at equal concentrations to form two pools. Library preparation and sequencing were conducted by Majorbio Bio-Pharm Technology Co., Ltd. (Shanghai, China). Qualified libraries were sequenced on an Illumina HiSeq platform, with an average sequencing depth of 10× for parental lines and 30× for progeny pools.

#### 2.4.2. Sequencing, Data Alignment, and Analysis

Raw sequencing reads were filtered to remove adapters, low-quality reads, and reads containing ambiguous bases (N). Filtered clean reads were aligned to the maize reference genome B73 (http://plants.ensembl.org/Zea_mays/Info/Index, (accessed on 17 October 2024)) using BWA software (version 1.0.6) [43]. Molecular markers were called using the GATK pipeline, which considers InDel realignment and mark duplication, and calls variants across all samples simultaneously through the Haplotype Caller program in GATK 4.1 [44]. Variants were filtered using standard hard filtering parameters according to the GATK Best Practices pipeline. Functional annotation of variants was performed using SnpEff software (version 5.1d) [45] and gene prediction information from the reference genome. The Euclidean distance (ED) and G′ value algorithms were used to identify SNPs associated with soluble sugar degradation rate. ED values were calculated for each locus based on sequencing depth differences between the two extreme pools. To reduce random fluctuations caused by individual variant sites, we calculated the fourth power of the original ED value and noise reduction was performed using a sliding window approach with a 1 Mb window size and 50 kb step size. For the G′ value association analysis, G-statistics were first calculated for each locus across the two pools. To reduce background noise and improve accuracy, smoothed G′ values were generated using a 1 Mb sliding window.

### 2.5. Analysis of the Expression for Candidate Genes

The public Phytozome database of maize was used for the expression analysis in different tissues (https://phytozome-next.jgi.doe.gov/, accessed on 22 January 2026). The expression levels from RNA-Seq data of candidate genes were calculated as the fragments per kilobase million reads (FPKM). The tissues included the seed, plant embryo, pericarp, endosperm, leaf, shoot apical meristem, anther, tassel inflorescence, ear inflorescence, silk, and root.

Quantitative reverse transcription PCR (qRT-PCR) was also employed for further validation in three lines from the F_6_ RIL population with different sugar degradation rates, including R218 (low degradation), R311 (moderate degradation) and R254 (high degradation). Total RNAs were extracted from kernels of these three lines at 0 h, 8 h and 72 h. The cDNA synthesis was performed using HiScript II Q Select RT SuperMix (Vazyme, Nanjing, China), and qRT-PCR was conducted on a SLAN-96P (Hongshitech Co., Ltd., Shanghai, China) using 2 × ChamQ Universal SYBR qPCR Master Mix (Vazyme Biotech Co., Ltd., Nanjing, China). The relative expression levels were calculated using the 2^−ΔCT^ method with *ZmActin* (NM_001155179.1) as the endogenous control [46]. Each sample included three biological replicates, and three technical replicates were analyzed for each gene. All the gene-specific primers used in this study are listed in Table S1.

### 2.6. Clustering Analysis of Paralog Genes

With the encoded protein of *Zm00001eb069070* as inquiry, the Blastp tool in NCBI (https://www.ncbi.nlm.nih.gov/, accessed on 22 January 2026) was used to search for paralogs in different species. The top 10 sequences with high similarity were employed for cluster analysis using the maximum likelihood method based on the JTT matrix-based model via MEGA 7.0 software [47]. The parameters used were as follows: test of phylogeny—bootstrap method; No. of bootstrap replications—1000; substitution type—amino acid; rates among sites—uniform rates.

### 2.7. Statistical Analysis

All data were subjected to one-way analysis of variance (ANOVA) using SPSS 21.0 software [48]. Means of treatments and genotypes were compared using Duncan’s multiple comparison test at a significance level of *p* < 0.01. Graphs were generated using Prism 10.0 (GraphPad, San Diego, CA, USA).

## 3. Results

### 3.1. Genetic Variation in Soluble Sugar Degradation Rate of Kernels in the RIL Population

**Figure 1:**
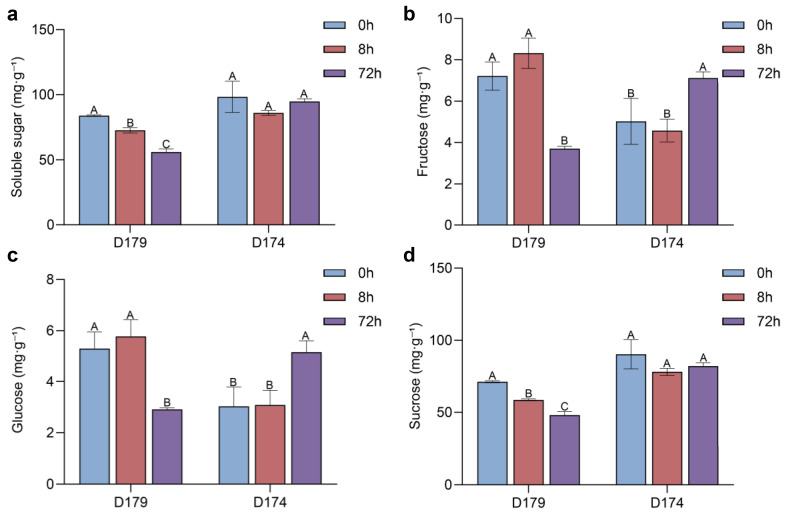
Changes in different soluble sugar contents of D179 and D174 within 72 h postharvest. (**a**) Changes in the total soluble sugar content. (**b**) Changes in fructose content of D174 and D179. (**c**) Changes in glucose content. (**d**) Changes in sucrose content of D174 and D179 within 72 h postharvest. The different soluble sugar contents at 0 h, 8 h, and 72 h postharvest are displayed in different colors. D179—high-degradation female parent; D174—low-degradation male parent. Different letters (A, B, C) above the bars indicate statistically significant differences at *p* < 0.01.

Soluble sugar content in the kernels is the primary factor controlling sweetness, and thus is critical to the eating quality of sweet corn [12,43]. The two inbred parental lines, D179 and D174, had total soluble sugar contents of 83.96 mg·g^−1^ and 98.32 mg·g^−1^ at 0 h post-harvest (*p* < 0.01), respectively, and both showed super sweetness (Figure 1a–d). After 72 h of storage, the soluble sugar content of D179 was 55.96 mg·g^−1^, with a reduction of 11.32 mg·g^−1^ and 28.00 mg·g^−1^ compared to 8 and 0 h, respectively. In contrast, the soluble sugar content of D174 at 72 h postharvest was 94.79 mg·g^−1^, showing no difference compared to 0 h (*p* > 0.01, Figure 1a). The soluble sugar degradation rate of strain D179 was significantly higher than that of D174 (*p* < 0.01, Figure 1a–d).

For the F_6_ RIL population, the average soluble sugar content of the 204 families at 0 h postharvest was 84.47 mg·g^−1^, with an average reduction of 13.79 mg·g^−1^ within 72 h. The variation range of soluble sugar reduction was −15.67 to 32.78 mg·g^−1^ (Figure S1). In the high-degradation pool, the average reduction in sucrose content reached 27.48 mg·g^−1^ within 72 h, representing a decrease of 36.65%, while no significant change was detected in the low-degradation pool (*p* < 0.01, Table S2). An evident bidirectional transgressive inheritance was observed for the soluble sugar degradation rate in the population (Figure S1, Table S2).

### 3.2. Mapping of Genes Related to Sugar Metabolism in Sweet Corn

#### 3.2.1. Quality Evaluation of BSA-Seq

BSA-seq was performed on four samples: two parental lines (D179 and D174) and two extreme phenotype pools (high- and low degradation). In total, 217.57 Gb raw sequencing data were obtained and then filtered using SOAPnuke software (version 2.1.9) to remove adapters, low-quality reads, and N-containing reads, yielding 214.97 Gb of high-quality clean data (Table 1). The GC content of each pool ranged from 44.79% to 45.72%. The proportion of bases with a quality value (Q) ≥ 30 in the sequencing libraries of D179, D174, the high-degradation pool, and the low-degradation pool was 95.45%, 95.37%, 95.11%, and 95.62% of the total bases, respectively (Table 1). These results indicate that the four samples had sufficient data volume, reliable sequencing quality, and normal GC distribution, making them suitable for subsequent analysis.

Clean filtered data were aligned to the maize reference genome (B73) using the BWA software (version 1.0.6, https://webblast.ipk-gatersleben.de/barley_ibsc/downloads/) (accessed on 17 October 2024). The average alignment efficiency was 99.71%, and the average coverage depth was 27.04×. The genome coverage ranged from 85.88% to 92.87% (Table 2). These results indicate that the BSA-seq data from different sample pools were of high quality and sufficient quantity, providing a solid foundation for the subsequent mapping analysis of sugar degradation traits.

#### 3.2.2. Detection and Annotation of SNPs and Indels

SNP detection was performed using the HaplotypeCaller method of GATK with strict filtering. The statistical results for the SNPs in the samples after alignment with the reference genome are shown in Table 3. A total of 9,582,677–11,963,692 SNPs were identified in the four samples. Among these, the highest number of mutations was located in the intergenic regions (8,420,445–10,548,554 SNPs), followed by the upstream regions (792,302–986,946 SNPs). In addition, 3331–3984 SNPs caused stop-gain codons and 585–700 SNPs caused stop-loss codons.

The indel detection results showed a total of 1,330,451–1,690,833 Indels across the four samples. Among these, 246,689–318,149 Indels were located in the upstream regions of the genes, 18,498–23,438 Indels caused frameshift mutations, 615–810 Indels resulted in stop-gain codons, and 319–400 Indels resulted in stop-loss codons (Table S3).

#### 3.2.3. Mining of Genetic Loci Associated with Postharvest Soluble Sugar Degradation in Sweet Corn by BSA-Seq

**Figure 2:**
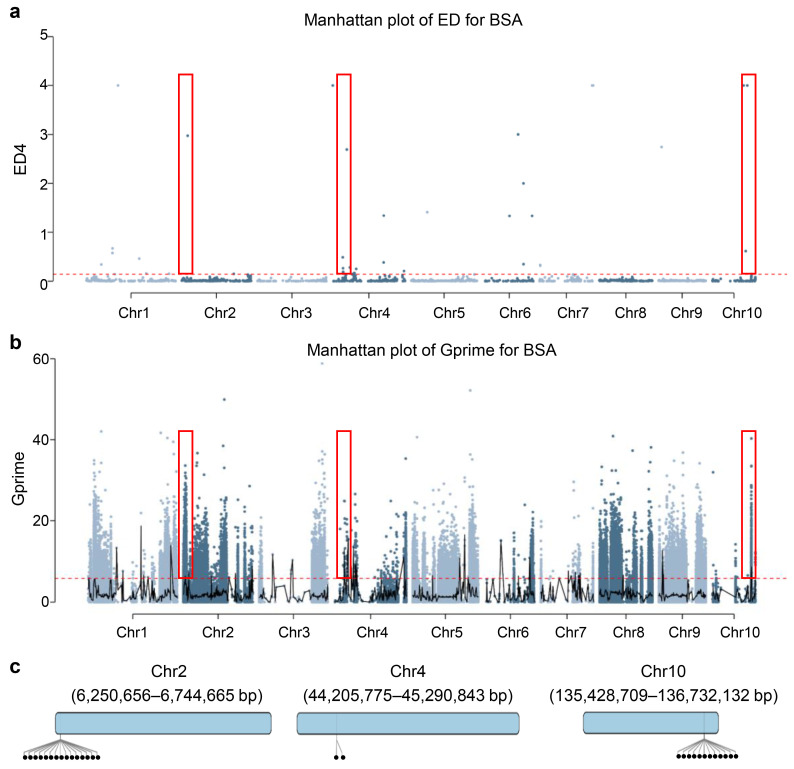
Distribution of association values on chromosomes identified using different methods. (**a**,**b**) Distribution of the ED (**a**) and G′ values (**b**). The *X*-axis represents the different chromosomes of maize, and the *Y*-axis represents the corresponding association values. The black and red lines represent the fitted values and association threshold, respectively. The consistent and reliable regions are highlighted in red boxes. (**c**) The consistent and reliable regions identified by ED and G′ values. The dots represent genes with functional annotations in the corresponding regions.

After strict quality filtering, a total of 8349 high-quality and reliable SNP loci were identified for association analyses. For the ED algorithm-based association analysis, candidate regions identified by each algorithm were required to contain at least 10 variant loci above the threshold to minimize bias from uneven marker distribution. To focus on functionally relevant regions, we performed strict variant filtering. All SNPs and InDels within each interval were annotated using SnpEff and classified into four impact categories: HIGH, MODERATE, LOW, and MODIFIER. We defined Effective SNPs and Effective InDels as those with HIGH or MODERATE impacts. Only intervals containing at least one effective variant were retained. As a result, using a 99.5% confidence threshold,13 intervals without effective variants were removed, and 7 significantly associated regions were mapped, which were distributed on chromosomes 1 (20,139,801–20,281,614 bp, 0.14 Mb; 26,887,016–27,434,800 bp, 0.55 Mb), chromosome 2 (6,250,656–6,744,665 bp, 0.49 Mb), 4 (44,205,775–45,290,843 bp, 1.08 Mb; 241,030,482–242,174,991 bp, 1.14 Mb), and chromosome 10 (135,428,709–136,732,132 bp, 1.30 Mb; 149,400,451–149,864,653 bp, 0.46 Mb) (Figure 2a and Table S4).

For the G′ value association analysis, with an association threshold set at 99.5%, ten associated regions were identified located on chromosome 1 (1,537,550–2,646,887 bp, 1.11 Mb), chromosome 10 (135,428,709–137,222,602 bp, 1.79 Mb), chromosome 2 (236,784,350–238,183,132 bp, 1.40 Mb; 6,061,733–7,046,050 bp, 0.98 Mb), chromosome 4 (35,128,883–37,543,654 bp, 2.41 Mb; 44,205,775–49,493,701 bp, 5.29 Mb; 73,096,581–76,483,355 bp, 3.39 Mb; 241,030,482–242,174,991 bp, 1.14 Mb), chromosome 7 (130,744,460–131,225,834 bp, 0.48 Mb; 132,838,844–133,484,141 bp, 0.65 Mb). The same criteria were applied to the G′ analysis; only intervals containing at least one effective variant were retained. As a result, 6 intervals were finally retained for further analysis (Figure 2b, Table S4).

By integrating the results from the two association methods (ED, and G′ value), three consistently and reliably associated regions were identified: (1) chromosome 4 (44,205,775–45,290,843 bp, 1.08 Mb), containing 32 genes; (2) chromosome 2 (6,250,656–6,744,665 bp, 0.49 Mb), containing 63 genes; and (3) chromosome 10 (135,428,709–136,732,132 bp, 1.30 Mb), containing 100 genes.

#### 3.2.4. Prediction and Validation of Candidate Genes

**Figure 3:**
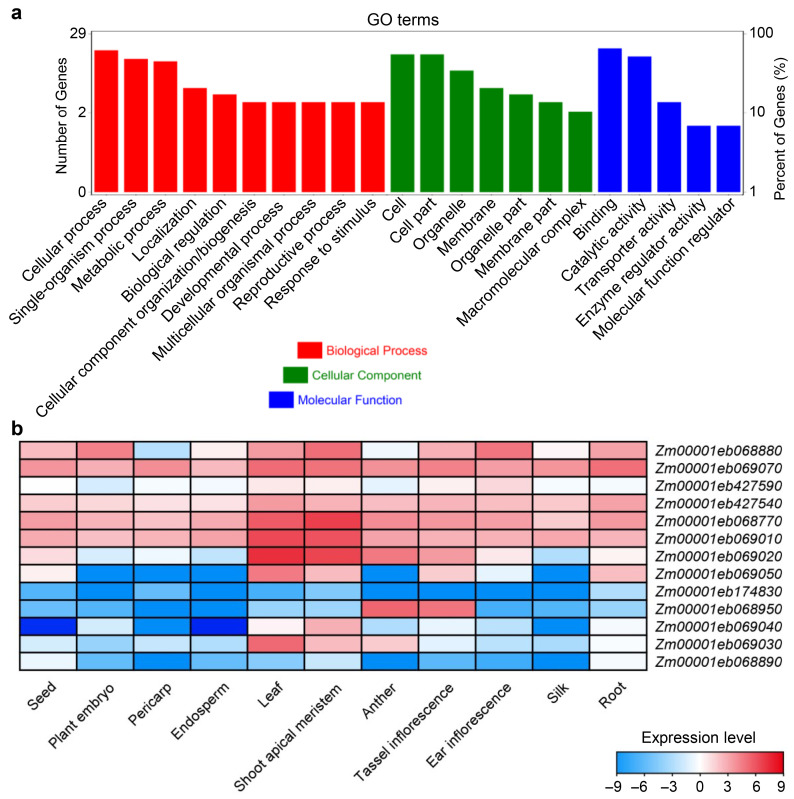
Identification of candidate genes in significantly associated regions. (**a**) GO annotation classification of genes at candidate intervals. The different bars represent the GO terms of biological process, cellular component, and molecular function. (**b**) Heatmap of expression levels for candidate genes in different tissues. The expression levels were calculated as log_2_(FPKM). FPKM, fragments per kilobase million reads.

Functional annotation of candidate genes within the three consistently associated regions was performed using the GO database. A total of 29 genes with annotated functional information were identified. These genes were classified into three functional categories: cellular components, molecular functions, and biological processes (Figure 3a). The results showed that the 29 genes were enriched in 22 biological processes. Notably, 19 genes were enriched in binding activity (GO: 0005488), and 18 genes were enriched in cellular processes (GO: 0009987), representing the most prominent functional categories. In the cellular component category, most candidate genes were involved in cell formation, cellular components, organelles, and membranes. Of particular relevance to sugar metabolism, several candidate genes were annotated with alcohol group-acceptor-type phosphotransferase activity (GO: 0016773), kinase activity (GO: 0016301), and carbohydrate metabolic processes (GO: 0005975). These functions are directly involved in sugar synthesis, starch storage and degradation, and sucrose transport—key processes underlying postharvest sugar dynamics in sweet corn.

The tissue expression characteristics of the 29 genes were analyzed using RNA-Seq data from the Phytozome database (https://phytozome-next.jgi.doe.gov/, accessed on 22 January 2026). Only 14 genes showed detectable expression in seed, plant embryo, pericarp, endosperm leaf, shoot apical meristem, anther, tassel inflorescence, ear inflorescence, silk, or root (FPKM > 1.0), whereas the remaining genes exhibited no or extremely low levels of expression (Figure 3b). Among them, *Zm00001eb068880*, *Zm00001eb069070*, *Zm00001eb427590*, *Zm00001eb427540*, *Zm00001eb068770*, *Zm00001eb069010*, *Zm00001eb069020*, and *Zm00001eb069050* displayed relatively high expression levels (FPKM > 1.0) in the seed or endosperm (Figure 3b). Gene functional annotation showed that only the first four genes (*Zm00001eb068880*, *Zm00001eb069070*, *Zm00001eb427590*, *Zm00001eb427540*) were involved in the process of sugar metabolism and might be related to sugar degradation, which could be further validated as candidate genes.

**Figure 4:**
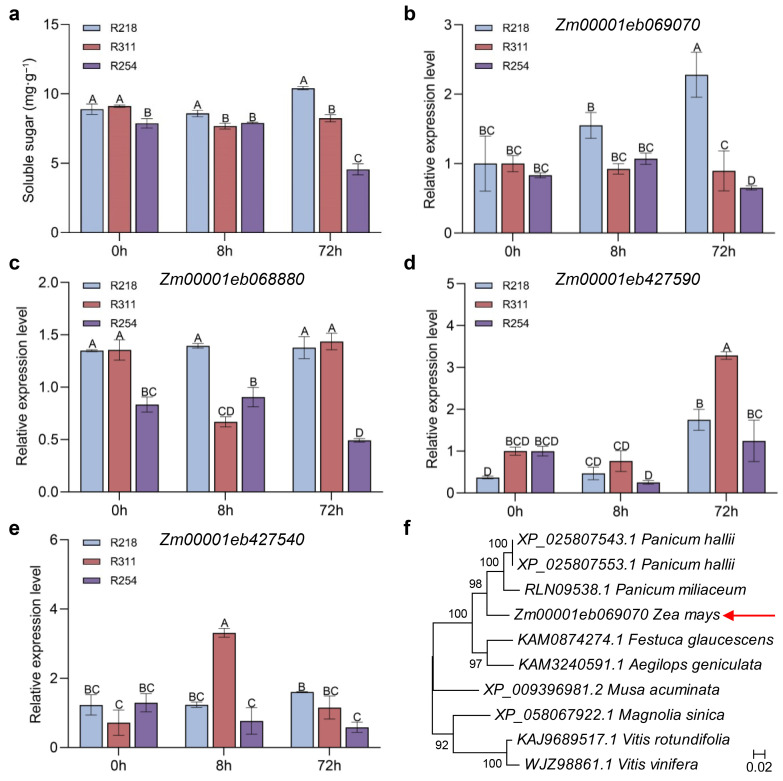
Analysis of soluble sugar contents and expression of candidate genes in R218, R311, and R254 within 72 h postharvest. (**a**) Changes in soluble sugar content. (**b**–**e**) The expression levels of candidate genes were detected via quantitative reverse transcription PCR (qRT-PCR). Three lines in the F_6_ RIL population were selected to detect the soluble sugar contents and gene expression, including R218 (low degradation), R311 (moderate degradation), and R254 (high degradation). (**f**) Clustering analysis of the paralogs of *Zm00001eb069070*. The top 10 sequences with high similarity to the encoded protein of *Zm00001eb069070* were downloaded from NCBI (https://www.ncbi.nlm.nih.gov/, accessed on 22 January 2026). Cluster analysis was performed using the maximum likelihood method based on the JTT matrix-based model via MEGA 7.0 software [47]. Different letters (A–D) above the bars indicate statistically significant differences at *p* < 0.01.

Three lines from the F_6_ RIL population had significant differences in sugar degradation rates (R218, low degradation; R311, moderate degradation; and R254, high degradation) (Figure 4a) and were selected for candidate-gene expression analysis by qRT-PCR. The results showed that the expression levels of *Zm00001eb069070*, *Zm00001eb427590*, and *Zm00001eb427540* showed no significant differences in expression among the three lines at 0 h postharvest (Figure 4b,d,e). *Zm00001eb069070* (Figure 4b) exhibited a significant decrease in expression from 0 h to 72 h, specifically in the high-degradation line R254. Additionally, *Zm00001eb069070* was consistently and significantly upregulated in the low-degradation line (R218) (*p* < 0.01). *Zm00001eb069070* was prioritized as the primary candidate gene for further investigation.

The Blastp tool in NCBI (https://www.ncbi.nlm.nih.gov/, accessed on 22 January 2026) was used to search the paralogs of the encoded protein of Zm00001eb069070. Among the top 10 sequences with high similarity in different species, three of them (XP_025807543.1 and XP_025807553.1 in *Panicum hallii*, RLN09538.1 in *Panicum miliaceum*) had the FGGY carbohydrate kinase domain, others were uncharacterized proteins. Domain analysis showed that the encoded protein of *Zm00001eb069070* contains an FGGY domain and belongs to the carbohydrate kinase family. We hypothesize that *Zm00001eb069070* may be closely associated with postharvest sugar metabolism in sweet corn. No relevant studies on this gene in maize have been reported, and functional validation of *Zm00001eb069070* will be conducted in subsequent research.

## 4. Discussion

Soluble sugars determine sweetness, which is a key quality evaluation indicator for sweet corn after harvesting. In sweet corn, sucrose is the main soluble sugar, but after harvesting, a high respiration metabolism can lead to sugar consumption, resulting in decreased sweetness and flavor deterioration [2,33,35,49]. Sweet corn exhibits a high respiration rate at harvest, and its vigorous metabolic activity leads to rapid water loss and sugar depletion, resulting in reduced sweetness and flavor deterioration [2,33,35]. Therefore, modifying storage conditions (e.g., low temperature, sealed packaging) is the most commonly used method to extend the postharvest shelf life of sweet corn [28,29].

In this study, we identified a natural mutant, DH174, which shows no significant postharvest soluble sugar degradation within 72 h (*p* < 0.01). Meanwhile, in the D179 line, the soluble sugar content decreased 28.00 mg·g^−1^ at 72 h postharvest (Figure 1a). Further research on the three main changes in soluble sugars revealed that this degradation is mainly triggered by the degradation of sucrose. In D179 and the high-degradation pool of F_6_ RIL population, the sucrose content decreased from 83.96 mg·g^−1^ to 55.96 mg·g^−1^ and from 74.97 mg·g^−1^ to 47.49 mg·g^−1^, respectively (Figure 1a, Table S2). Therefore, the observed reduction in the relative proportion of sucrose is the core factor underlying postharvest quality deterioration. This sucrose-dominated degradation pattern is aligned with previous studies [50,51] showing that sucrose degradation is the major cause of sweetness loss in stored sweet corn, further supporting the critical role of sucrose metabolism in postharvest quality retention. The discovery of this mutant provides research material for investigating the regulatory mechanisms of sugar metabolism, and more importantly, a valuable genetic resource for breeding improved sweet corn varieties.

The sugar metabolism regulatory network is complex and regulated by multiple genes [52,53]. The bidirectional transgressive inheritance observed for soluble sugar degradation rate in the F_6_ RIL population (Figure 1 and Figure S1, Table S2) further confirms that postharvest sugar metabolism is a complex quantitative trait controlled by multiple genetic loci. Some RIL families exhibited higher sugar degradation rates than the high-degradation parent D179 (sugar reduction > 28.00 mg·g^−1^, maximum 32.78 mg·g^−1^), while others showed lower rates (even negative reduction values, i.e., increased soluble sugar content) than the low-degradation parent D174 (sugar reduction < 3.52 mg·g^−1^, minimum −15.67 mg·g^−1^). This transgressive phenomenon may arise from recombination and reassortment of alleles from both parents during meiosis: D179 likely carries dominant alleles promoting sucrose hydrolysis, while D174 harbors recessive alleles inhibiting degradation. The bidirectional transgressive inheritance observed for soluble sugar degradation rate in the RIL population (Figure S1) further confirms that postharvest sugar metabolism is a complex quantitative trait controlled by multiple genetic loci. Some RIL families exhibited higher sugar degradation rates than the high-degradation parent D179 (sugar reduction >28.00 mg·g^−1^, maximum 32.78 mg·g^−1^), while others showed lower rates (even negative reduction values, i.e., increased soluble sugar content) than the low-degradation parent D174 (sugar reduction <3.52 mg·g^−1^, minimum −15.67 mg·g^−1^). This transgressive phenomenon may arise from recombination and reassortment of alleles from both parents during meiosis: D179 likely carries dominant alleles promoting sucrose hydrolysis and starch synthesis, while D174 harbors recessive alleles inhibiting degradation.

Sweet corn is the result of mutations in genes involved in the starch biosynthesis pathway, and these mutations modify carbohydrate composition by increasing sugar content in the endosperm while reducing starch accumulation [24]. Mutants such as *brittle1* (*bt1*), *brittle2* (*bt2*), and *shrunken2* (*sh2*) accumulate four to eight times higher total sugar than normal corn at harvest stage by sacrificing starch synthesis, while *amylose extender1* (*ae1*), *dull1* (*du1*), and *waxy1* (*wx1*) alter polysaccharide composition with milder sugar increases alone but achieve comparable sugar levels via double/triple combinations [54]. The *sh2* encodes the large subunit of ADP–glucose pyrophosphorylase (AGPase), initiating the committed step of starch synthesis [24]. Li et al. [55] de novo assembled a cultivated sweet corn genome and resequenced 295 diverse sweet corn inbred lines. By combining genetic, metabolite and expression profiling methodologies, three novel genes (*ZmAPS1*, *ZmSK1,* and *ZmCRR5*) associated with flavor and consumer preference were identified. Specifically, *ZmAPS1* regulates adenosine metabolism, influencing the synthesis of flavor precursors; *ZmSK1* affects quinic acid synthesis, which is closely associated with the aroma of sweet corn, and *ZmCRR5* modulates fructose accumulation, playing a critical role in balancing sweetness and yield. Using gene editing technology, they confirmed that these genes regulate different metabolic pathways, which collectively control the edible quality of sweet corn. Transcriptomics and metabolomics were employed to investigate the quality changes in different sweet corn varieties. It was found [2] that there were differences in the expression levels of *β-fructofuranosidase* (*INVA*, *Zm00001eb24280*, involved in sucrose metabolism, hydrolyzes sucrose into glucose and fructose), *BMY* (*Zm00001eb323450*, involved in starch degradation), as well as starch synthase 4 (SS4, *Zm00001eb353810*) and starch synthase 5 (*SS5*, *Zm00001eb351550*) (involved in both starch and sucrose metabolism pathways), which further led to varying degrees of postharvest quality deterioration among different varieties in terms of appearance, sugar composition, texture, and flavor.

In this study, by integrating BSA-seq, published transcriptome data, gene functional annotations, and qRT-PCR analysis, *Zm00001eb069070* was identified as a potential candidate gene regulating postharvest sweetness reduction in sweet corn. *Zm00001eb069070* contains an FGGY domain and belongs to the carbohydrate kinase family. To date, few relevant research reports on *Zm00001eb069070* have been found in maize or other cereal crops, suggesting it may be a novel regulatory gene for postharvest sugar metabolism in sweet corn. Further functional verification is required to confirm its specific role in sucrose degradation and postharvest sweetness retention, including gene overexpression, knockout, and enzyme activity assays.

## 5. Conclusions

This study identifies key genetic loci and a candidate gene associated with soluble sugar dynamics in sweet corn kernels during postharvest storage. Soluble sugar content gradually declined during prolonged storage, consistent with typical postharvest physiological changes in sweet corn. Using BSA-seq, three consistent genomic regions significantly associated with postharvest sugar degradation were identified: chromosome 4 (44,205,775–45,290,843 bp), chromosome 2 (6,250,656–6,744,665 bp), and chromosome 10 (135,428,709–136,732,132 bp). Based on published transcriptome profiling, gene annotation, and qRT-PCR validation, *Zm00001eb069070* was identified as a promising candidate gene potentially involved in regulating postharvest sugar metabolism in sweet corn. These findings provide novel genetic resources and a theoretical foundation for understanding the genetic basis of soluble sugar degradation in postharvest sweet corn, which may facilitate molecular breeding aimed at improving storage stability and shelf life. Future studies are needed to validate the precise biological function of *Zm00001eb069070* and to explore its regulatory mechanism in sugar metabolism and postharvest preservation, which could support the development of sweet corn varieties with extended shelf life and improved quality.

## Figures and Tables

**Table 1:** Statistical analysis of sequencing data quality evaluation.

Samples	Raw Reads (bp)	Raw Bases (bp)	Clean Reads (bp)	Clean Bases (bp)	Clean GC (%)	Clean Q30 (%)
D179	89,637,444	27,070,508,088	177,895,902	26,777,569,770	45.65	96.05
D174	86,738,930	26,195,156,860	172,093,802	25,873,716,674	45.10	96.06
HL	254,355,743	76,815,434,386	574,316,392	86,393,081,415	45.72	95.82
SL	289,685,454	87,485,007,108	504,655,312	75,923,082,583	44.79	96.28

Note: D179—high-degradation female parent; D174—low-degradation male parent; HL—high-degradation pool; SL—low-degradation pool. The same applies hereinafter.

**Table 2:** Statistical analysis of alignment results with the reference genome.

Samples	Mapped Ratio (%)	Proper Ratio (%)	Insert Size	Real Depth	Coverage (%) (≥1×)	Coverage (%) (≥4×)
D179	99.71	91.62	399	14.25	85.88	74.63
D174	99.72	91.78	402	13.72	86.20	74.25
HL	99.70	91.25	419	42.5	92.87	87.45
SL	99.72	92.30	305	37.68	92.09	86.16

**Table 3:** Number and quality analysis of SNPs identified by bulked segregant analysis sequencing (BSA-seq) in parental lines and two pools.

Item	D179	D174	HL	SL
Intergenic	8,420,445	8,562,833	10,516,306	10,548,554
Intronic	1,847,340	1,925,213	2,266,998	2,269,944
Upstream	792,302	817,899	986,440	986,946
Downstream	760,458	793,082	942,203	942,461
Splicing	1230	1272	1430	1453
Start-lost	476	483	563	561
Synonymous	224,379	232,751	273,421	273,142
Stop-gain	3331	3416	3984	3972
Stop-lost	602	585	700	695
Total number	9,582,677	9,769,451	11,930,403	11,963,692

## Data Availability

The data presented in this study are openly available on the phytozome website or other websites.

## Supplementary Materials

The following supporting information can be downloaded at https://www.mdpi.com/article/10.3390/genes17030291/s1, Figure S1: Frequency distribution of soluble sugar content (a) and sugar degradation (b) in the recombinant inbred line (RIL) population; Table S1. Primers used in this study for Quantitative Real-Time PCR; Table S2: The changes in soluble sugar in HL and SL groups within 72 h; Table S3: Number and quality analysis of Indels identified by BSA-seq in parental lines and two corresponding pools; Table S4: Genetic loci of sugar-reduction traits in kernels via different methods.

## Abbreviations

The following abbreviations are used in this manuscript:

Inv	Invertase
SPS	Sucrose Phosphate Synthase
SUS	Sucrose Synthase
FRK	Fructokinase
HXK	Hexokinase
F6P	Fructose-6-phosphate
G6P	Glucose-6-phosphate
UDP-glucose	Uridine Diphosphate Glucose
SPP	Sucrose-Phosphate Phosphatase
BSA	Bulked Segregation Analysis
RNA-seq	RNA sequencing
BSA-seq	Bulked Segregation Analysis Sequencing
RIL	Recombinant Inbred Line
DAP	Days after Pollination
HPLC-ELSD	High-Performance Liquid Chromatography–Evaporative Light Scattering Detection
BWA	Burrows–Wheeler Aligner
GATK	GATK
Indels	Insertions/Deletions
SNPs	Single-Nucleotide Polymorphisms
ED	Euclidean Distance
SnpEff	SNP Effect Predictor
FPKM	Fragments Per Kilobase Million Reads
qRT-PCR	Quantitative Real-Time PCR
Blastp	Basic Local Alignment Search Tool for proteins
NCBI	National Center for Biotechnology Information
ANOVA	Analysis of Variance
SPSS	Statistical Product and Service Solutions
SOAPnuke	SOAPnuke sequencing data filtering software
FGGY	FGGY carbohydrate kinase domain
ADP–glucose	Adenosine Diphosphate Glucose
AGPase	ADP–glucose pyrophosphorylase
SS4	Starch Synthase 4
SS5	Starch Synthase 5
