# Supplementary material for: Identification of Candidate Gene Controlling Soluble Sugar Degradation During Postharvest Storage of Sweet Corn Based on BSA-Seq
Source: Genes (Basel). 2026 Feb 27;17(3):291. doi: 10.3390/genes17030291 (PMC13025937; doi:10.3390/genes17030291)
Supplement: Supplementary file 1 [file genes-17-00291-s001.zip › genes-4140921-supplementary.pdf]

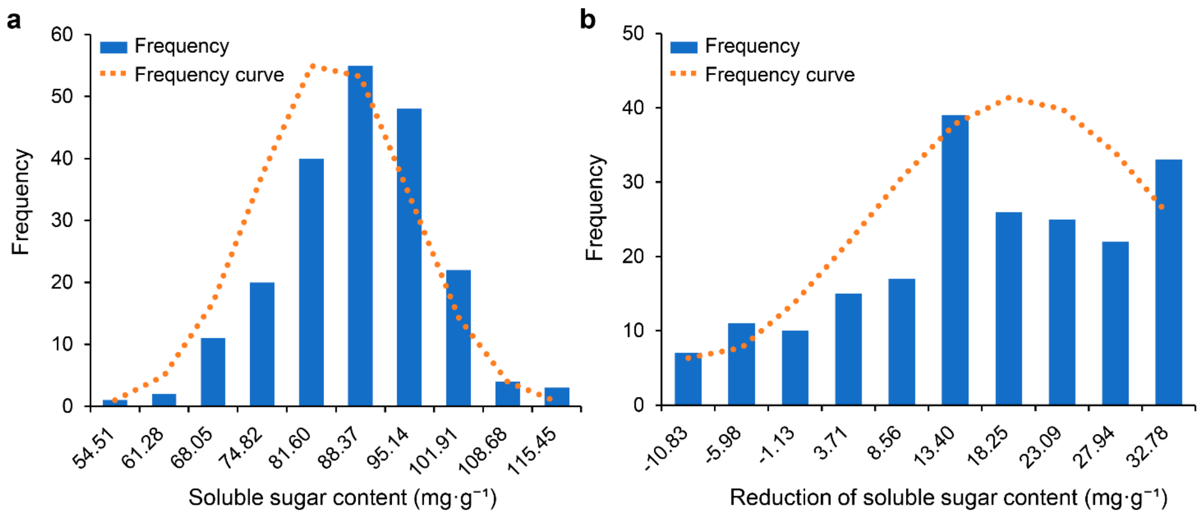

Figure S1. Frequency distribution of soluble sugar content (a) and sugar degradation (b) in the recombinant inbred line (RIL) population. The X-axis represents the soluble sugar content at 0 h post-harvest (a) and reduction of soluble sugar content within 72 h post-harvest (b), respectively, and the Y-axis represents the corresponding frequency. The blue bars and orange lines represent the frequency and frequency curve, respectively.

Table S1. Primers used in this study for Quantitative Real-Time PCR.

Gene	Forward sequence (5'-3')	Reverse sequence (5'-3')
ZmActin	ACTGAAGTACCCGATTGAG	CGTTGTAGAAGGTGTGATG
Zm00001eb068880	CATTACTTCTTCAGCATCA	CAACCAACTTCATTCCTAA
Zm00001eb069070	GGCTTGACACTTGATACA	ACAATGCTCCACAATATGA
Zm00001eb427590	CAATGTAACCAGTATGTC	TCAGAGAATGTAATAGGAT
Zm00001eb427540	GATGTTGATGATGTTGAT	GTTATTACTGTGCTACTG
Zm00001eb174790	TTACAGACGGCTACATCA	TCGGCAATATCAGAGGAT

Table S2. The changes of soluble sugar in HL and SL groups within 72 hours.

Soluble sugar content ($mg \cdot g^{-1}$)	HL		SL	
	0h	72h	0h	72h
Soluble sugar content	85.25±9.28A	61.95±12.12B	82.05±11.22B	90.27±12.28A
Fructose content	3.99±0.96B	5.95±0.74A	3.41±1.0B	6.66±2.22A
Glucose content	5.42±1.17B	7.26±1.61A	4.89±1.16B	8.03±2.36A
Sucrose content	74.97±8.72A	47.49±1.54B	73.46±10.54A	74.36±11.11A

Note: HL, high-degradation pool; SL, low-degradation pool.

Table S3. Number and quality analysis of Indels identified by BSA-seq in parental lines and two corresponding pools.

Item	D179	D174	HL	SL
Intergenic	1,029,812	1,055,786	1,339,866	1,339,656
Intronic	480,428	502,207	608,958	609,247
Upstream	246,689	255,205	318,149	317,437
Downstream	231,912	243,102	300,770	299,901
Splicing	15,571	16,396	19,353	19,371
Start-lost	546	538	691	693
Frame-shift	18,498	18,898	23,438	23,323
Stop-gain	615	679	810	780
Stop-lost	319	332	391	400
Total number	1,330,451	1,364,740	1,690,490	1,690,833

Note: D179, high-degradation female parent; D174, low-degradation male parent; HL, high-degradation pool; SL, low-degradation pool.

Table S4. Genetic loci of sugar-reduction traits in kernels via different methods.

Chromosome	Start (bp)	End (bp)	Interval size (Mb)	Gene numbers	Association analysis methods
Chr1	20,139,801	20,281,614	0.14	17	Euclidean
Chr1	26,887,016	27,434,800	0.55	35	Euclidean
Chr2	6,250,656	6,744,665	0.49	63	Euclidean
Chr4	241,030,482	242,174,991	1.14	81	Euclidean
Chr4	44,205,775	45,290,843	1.08	32	Euclidean
Chr10	135,428,709	136,732,132	1.30	100	Euclidean
Chr10	149,400,451	149,864,653	0.46	84	Euclidean
Chr1	1,537,550	2,646,887	1.11	66	G' value
Chr2	236,784,350	238,183,132	1.40	76	G' value
Chr2	6,061,733	7,046,050	0.98	113	G' value
Chr4	241,030,482	242,174,991	1.14	81	G' value
Chr4	44,205,775	49,493,701	5.29	122	G' value
Chr10	135,428,709	137,222,602	1.79	122	G' value
